# Editorial: Optimal physical activity across the lifespan for people of all abilities

**DOI:** 10.3389/fspor.2025.1645447

**Published:** 2025-06-26

**Authors:** David A. Hart, Ronald F. Zernicke

**Affiliations:** ^1^ Department of Surgery, Faculty of Kinesiology, McCaig Institute for Bone & Joint Health, University of Calgary, Calgary, AB, Canada; ^2^ Department of Orthopaedic Surgery, Michigan Medicine, School of Kinesiology, University of Michigan, Ann Arbor, MI, United States; ^3^ Department of Biomedical Engineering, University of Michigan, Ann Arbor, MI, United States

**Keywords:** physical activity, exercise, MSK health, cardiovascular health, across the lifespan

**Editorial on the Research Topic**
Optimal physical activity across the lifespan for people of all abilities

Humans, like all current species, evolved within the context of the boundary conditions of Earth, including its 1 g gravity. In that context, we evolved to move, making us a dynamic species that subscribes to the “use it or lose it” principle for many of our tissues (1). Humans depend on motion and navigation through the environment to survive, and thus, depend on mechanical loading of our tissues to maintain the integrity of nearly all our physiological systems, as evidenced by the adverse effects of prolonged exposure to microgravity in space, following prolonged bed rest, and generalized inactivity. Inactivity and its accompanying diminished loading conditions on physiological systems pose a threat to health, and exercise is required to maintain and optimize health worldwide [reviewed in (2–5)]. Thus, *Homo sapiens* likely require constant and repetitive loading of our tissues during the day, adequate nutrition, and periods of rest (i.e., sleep) (6) to optimize quality of life and achieve wellness throughout life.

Much of the focus on the benefits of exercise has been on elite athletes who push the boundaries of our genetic inheritance to achieve higher and higher levels of accomplishment. Clearly, the abilities of Olympic athletes are examples of this, as are those of other amateur and professional athletes. These abilities are contingent upon intense exercise and training programs, along with optimal mental health, and they do provide new insights about the upper limits of human capabilities. However, they can also have consequences such as injuries to essential physiological systems, such as the musculoskeletal (MSK) system. In addition, focusing only on the extremes of exercise and functionality can cause us to lose sight of the fundamental premise that loading of our tissues—particularly those of the MSK and cardiovascular systems—is essential for health, and thus important for individuals of all ages and abilities. While all humans benefit from physical activity, women and men and different ethnicities may experience risks and benefits (7), so “one size may not fit all”.

In recent years, there has been a shift in many “modern” occupations from being physically demanding to involving prolonged periods of “inactivity” while sitting at a desk in front of a computer screen [reviewed in (8)]. For example, children and adolescents sit in school for much of the day and in front of their electronic devices, leading to extended periods of inactivity in otherwise healthy populations that are growing and maturing, and thus setting a suboptimal “baseline” for several physiological systems during a critical phase of life (9, 10)! In addition to these populations, others of different ages may have compromised abilities to perform exercise as a result of limited access to programs, physical limitations due to injury, developmental deficits, and/or chronic diseases or complications from lifestyle choices and their consequences. These populations can still benefit from tailored, adaptive exercise programs that offer aerobic and/or resistive exercises ± augmentation, which allow for maintenance of essential physiological systems.

To address many of the issues and challenges to meet those needs in populations across a wide spectrum of abilities, this Research Topic of articles was conceived and compiled. It consists of both original research reports and reviews. The Research Topic of articles includes 11 peer-reviewed and accepted submissions that provide excellent examples of how physical activity programs can benefit specific populations ranging from the young to the elderly with or without specific limitations, along with those individuals who are motivated to achieve excellence despite perceived limitations, as seen in the Paralympics.

The spectrum of articles in the Research topic addresses several conditions, diseases, and circumstances that impact a wide variety of populations. These include different types of exercise programs and activities that target specific populations such as neurodivergent children (Sapre et al.), patients with Parkinson's disease (McKee et al.), individuals with spinal cord injuries (Martinez et al.), and elderly patients with changes in cognition (MacDonald et al.). Other articles assess the efficacy of physical activity programs (Muñoz-Cofré et al.) and the use of tele-exercise programs for individuals with difficulties accessing regular programs (Garrido et al.). The effectiveness of cycling as a means to achieving aerobic fitness is addressed (Mosser et al.), as is the use of neuromuscular electrical stimulation to offer potential benefits to various populations (Ackermann et al.). Other articles in this Research Topic provide insights into how physical activity may benefit specific populations, such as military veterans (Tinney and Nguyen) and those individuals affected by the consequences of infectious diseases, such as COVID-19 (Opielinski et al.). Finally, an article by Bonnevie-Svendsen et al. provides insights into some recently identified biomarkers in triathletes. Building on these results, it would be interesting in the future to investigate whether similar or different biomarkers are also identified as determinants of the efficacy of physical activity programs for the specific populations that are the focus of other articles in this Research Topic.

In summary, this Research topic focuses on addressing the need for and benefits of physical activity in populations across a wide spectrum of abilities. This Research Topic complements an earlier Research Topic that we edited, which focused on the benefits of physical activity for those aspiring to achieve at the highest levels (11). We intend for this Research Topic to highlight the need for and benefits of physical activity for *Homo sapiens* of all abilities to engage and perhaps to provide inspiration for some populations not covered in this Research Topic to develop and implement specialized physical activity programs, providing an even broader spectrum of positive health benefits for individuals whose abilities are limited or challenged by circumstances.
